# Urinary pH in calcium oxalate stone formers: does it matter?

**DOI:** 10.1590/1678-4685-JBN-2018-00010002

**Published:** 2018-04-09

**Authors:** Mauricio Carvalho

**Affiliations:** 1 Universidade Federal do Paraná, Departamento de Clínica Médica, Hospital de Clínicas, Curitiba, PR, Brasil.; 2 Pontifícia Universidade Católica do Paraná, Escola de Medicina, Curitiba, PR, Brasil.

Urinary pH is a major determinant for kidney stone formation. Supersaturation of calcium phosphate increases rapidly as urine pH rises above 6. The prevalence of calcium phosphate stones has increased recently and urinary conditions that favor the formation of these stones are the combination of hypercalciuria and hypocitraturia in alkaline urine. Some patients may have complete or incomplete distal renal tubular acidosis (dRTA) characterized by hyperchloremic acidosis (incomplete dRTA does not manifest metabolic acidosis under basal conditions), hypocitraturia, and high urine pH. The use of carbonic anhydrase inhibitors such as acetazolamide and topiramate leads to a similar scenario.^1^

A low urine pH is the most important factor leading to uric acid stone formation. Uric acid is a weak organic acid with a pKa of 5.5. At such low pH, soluble uric acid crystallizes. Quantitatively, with urine pH of 5.3 and urate excretion of 800 mg/day, precipitation of uric acid would likely occur with a daily urine volume that is as high as two liters.^2^ In obese patients, because of hyperinsulinemia and insulin resistance related to visceral obesity, dysfunction in ammonium excretion and urine acidification in the proximal tubule cause low urinary pH.^3^ In fact, in recent years, kidney stone formers present with high BMI (overweight or obesity), increased waist circumference, and high body fat percentage. These findings might be associated with increased calcium, oxalate, and uric acid excretion, thus increasing the risk of kidney stone formation.^4^

It is well known that most calcium stones are composed primarily of calcium oxalate (~80%). The role of urine pH in the formation of calcium oxalate stones is highly controversial. Most authors found that calcium oxalate stones could form in any urine pH. The benefit of increasing urine pH in calcium oxalate stone formers with low urine pH and normal urinary citrate is uncertain.

In a study published in this issue of BJN, Tessaro et al. analyzed the influence of nutritional status, laboratorial parameters, and dietary patterns on urinary acid excretion in calcium stone formers. They concluded that the endogenous production of organic acids and not an acidogenic diet was an independent predictor factor for lower urinary pH levels in calcium stone formers.^5^ In addition, hypercalciuric and/or hyperuricosuric patients presented higher organic acid levels and lower urinary pH.

Despite the limitations of a retrospective study and the lack of measurement of some urinary components (like sulfate, a direct marker of acid intake, or ammonium), the study brings to light several questions. Theoretically, a low urinary pH can be caused by increased base loss, increased acid intake (high consumption of animal protein), increased endogenous acid production, and decreased urinary ammonium. If increased endogenous acid production is the cause for the lower urinary pH found in hypercalciuric stone formers as reported here, the pertinent questions are: 1) what is the mechanism? 2) Is it clinically relevant?

Insulin resistance and obesity are associated with increased endogenous acid production and may be the most obvious explanation. However, is it possible that urinary calcium per se can stimulate urinary acidification? Double KO mice lacking the TRPV5 calcium channel (the major pathway for transcellular calcium reabsorption in the late distal tubule) and B1 H+-ATPase subunit (part of the cytosolic H+-ATPase pump from intercalated cells and required for maximal urinary acidification) present with massive increase in calciuria and phosphaturia and more alkaline urine than wild-type mice. The result is massive hydronephrosis and kidney stones.^6^ Defensive mechanisms may protect from kidney stone formation in conditions such as hypercalciuria where high luminal calcium concentrations stimulate urinary acidification and reduce urinary concentration via a calcium-sensing receptor, resulting in the excretion of acidic and diluted urine.^7^ Whether these protective mechanisms can be translated from mice to humans is uncertain.^8^

Finally, there is almost a consensus that calcium oxalate supersaturation is independent of urine pH. Nonetheless, a recently experimental work revealed that CaOx monohydrate was crystallized with greatest size, number, and total mass at pH 4.0 and least crystallized at pH 8.0. Fourier-transform infrared (FT-IR) spectroscopy confirmed the morphological study. Moreover, the crystal-cell adhesion assay showed highest crystal-cell adhesion at the most acidic pH.^9^ Unquestionably, further studies with large clinical databases providing population characteristics, identifying risk factors, and developing prognostic models are necessary to confirm the clinical relevance of these findings.^10^
